# A Clinical Study on Mobility Outcomes After Surgical Fixation of Intertrochanteric and Shaft Femur Fractures

**DOI:** 10.7759/cureus.94503

**Published:** 2025-10-13

**Authors:** Usman Ismail Khalid, Kainat Zafar, Syed Ahmad Bilal Bukhari

**Affiliations:** 1 Trauma and Orthopaedics, Royal Victoria Hospital, Belfast, GBR; 2 Pathology, Dr. Ziauddin Hospital, Karachi, Karachi, PAK; 3 Orthopaedic Surgery, Akhtar Saeed Trust Hospital EME Sector, Lahore, PAK

**Keywords:** femoral fractures, fracture fixation, internal, mobility limitation, postoperative complications, treatment outcome

## Abstract

Background: Femoral fractures are a major orthopedic burden with comprehensive implications on both the mobility and health care resources of the patient. Early rate of recovery and functional locomotion is the most significant in its ability to reduce hospitalization and maximize postoperative quality of life after surgical repair. This study was performed to compare early postoperative mobility, complications, and length of stays of intertrochanteric and femur shaft fracture patients undergoing surgery.

Methods: The prospective study consisted of 90 adults with isolated intertrochanteric or shaft femur fractures in a tertiary orthopedic unit over 18 months. The patients were assigned into two groups determined by the type of fracture: intertrochanteric fractures (n = 45) were undergoing treatment with dynamic hip screws, and shaft femur (n = 45) with intramedullary nailing. The PROMIS Physical Function (PROMIS-PF) short form and Timed Up-and-Go (TUG) tests were used to evaluate mobility after three and six months of the surgery. Hospital stays and complications were also noted. The statistical analysis was conducted by SPSS (IBM Corp., Armonk, NY, USA) using chi-square, independent t-tests, and the Pearson correlation method. Statistical significance at p < 0.05 was considered significant.

Results: Ninety patients were taken (45 each group) with a mean age of 63.8 years, where the intertrochanteric group was older (66.1 vs. 61.5 years, p=0.038) but similar in sex and comorbidities. The difference in the mean PROMIS-PF 10a scores between intertrochanteric fracture and shaft fracture was significant after three and six months of follow-up (58.4 vs. 52.7, p=0.003, and 66.3 vs. 60.6, p=0.004). The TUG test results indicated quicker mobility times in the intertrochanteric group after three and six months (p=0.012 and p=0.007, respectively). The rates of complications were not different across the groups (five (11.1%) vs. six (13.3%), p=0.743), mainly postoperative wound infections, transfusion requirements, and delayed union. Intertrochanteric fracture patients spent less time in the hospital compared to shaft fracture patients; 7.2 ± 2.1 versus 9.0 ± 3.4 days (p=0.001).

Conclusion: Better early mobility and reduced hospitalization rates are associated with the surgical fixation of intertrochanteric femur fractures than shaft femur fractures. These findings suggest more specific rehabilitation guidelines and fracture-type-dependent counseling for patients.

## Introduction

Femoral fractures are a major health issue worldwide, as the number of people affected by them creates a heavy burden on emergency care and rehabilitation [1]. The incidence of intertrochanteric femur fractures continues to rise in the elderly due to prevalent osteoporosis and low-energy falls, which frequently lead to a prolonged recovery process and loss of functional independence [2]. Conversely, shaft femur fractures commonly affect younger individuals with high-energy traumas, which limits their early mobilization regardless of recent surgical fixation [3]. Dynamic hip screw fixation for intertrochanteric fractures and intramedullary nailing of shaft fracture represent standard methods of fixation, which have been optimized to provide better outcomes; however, gaps in the recovery dynamics remain [4]. The primary goal is to restore mobility as early as possible to reduce complications, improve quality of life, and limit hospital stays [5].

According to emerging studies, functional recovery depends on various factors, including the type of fracture, patient characteristics, and surgical approach [6]. Nevertheless, the existing data comparing mobility results of these fractured types are comparatively scarce, particularly in a similar clinical environment [7]. The absence of clear evidence does not allow for establishing tailored rehabilitation pathways, and clinicians define realistic expectations among patients with various femoral fractures [8]. Further research is required to determine the substantial impact of fractured location on postoperative mobility and recovery time, which may also help in counseling patients.

The objective of this study was to compare early postoperative mobility between patients who underwent surgical fixation of intertrochanteric and shaft femur fractures. It further aimed to determine the rates of complications and hospital stay between these two groups. Moreover, the purpose was also to establish evidence to direct the creation of individualized rehabilitation strategies.

## Materials and methods

This was a prospective observational study with a duration of 18 months conducted in the trauma unit in a tertiary healthcare setting (Ref: 4123) from October 2021 to March 2023 and included an informed consent process. A consecutive sampling technique was used to recruit patients by considering all eligible patients who approached the facility during the study period to participate. A sample size was calculated using OpenEpi version 3.0.0 (released 2013, Atlanta, GA, USA) of a minimum of 45 patients per group with an alpha of 0.05, and a power of 80% [9]. This sample size seemed sufficient to detect meaningful differences in Patient-Reported Outcomes Measurement Information System Physical Function Short Form (PROMIS PF-10a) scores, while balancing all requirements. The inclusion criteria were adults aged 18 years and above with an intertrochanteric or shaft femur fracture, requiring surgical fixation. Patients with pathological fractures, polytrauma, pre-existing mobility deficits, or non-consent were excluded from the study.

Participants were categorized into two groups according to the fracture type: the intertrochanteric group (received dynamic hip screwing fixation) and the femoral shaft group (received antegrade intramedullary nailing) by an experienced orthopedic surgeon according to known protocols. Compliance with the recommended surgical procedure and post-operative management was achieved by implementing operative auditing procedures and uniform rehabilitation protocols. After three and six months of surgery, publicly open-access resources, including the PROMIS PF-10a (v2.0; HealthMeasures, Chicago, IL, USA; used with official items and scoring instructions, without modification) developed by the PROMIS Health Organization, and the Timed Up-and-Go (TUG), were used to ascertain the mobility outcomes [10,11]. Both tools were utilized under the available terms of usage. Higher PROMIS-PF 10a scores and lower TUG scores indicated better mobility. Secondary outcome measures included complication rates and length of stay.

Analysis of data was conducted by SPSS version 26.0 (released 2019, IBM Corp., Armonk, NY, USA). Continuous variables were analyzed using independent t-tests, and categorical data using chi-square tests. The Pearson correlation method was used to analyze the relationship between age and mobility outcomes. A p-value of < 0.05 was considered statistically significant.

## Results

The purpose of this study was to contrast mobility outcomes and hospital stay following surgical fixation. The outcomes of a surgery in 90 patients with femur fractures were examined in equal numbers of intertrochanteric and shaft fracture groups. The groups were similar in sex and comorbidities, with the intertrochanteric group being slightly older (p=0.038). The time between the injury and the surgery was not significantly different between groups. Demographic and clinical characteristics of study participants are summarized in Table 1.

**Table 1:** Baseline Demographic and Clinical Characteristics of Study Participants n = Number of participants, SD = Standard Deviation, % = Percentage, * = Significance at p-value <0.05

Parameter	Intertrochanteric Group (n=45)	Shaft Femur Group (n=45)	Test Used	Test Value	Significance (p-value)
Mean Age (years) Mean ± SD	66.1 ± 10.2	61.5 ± 12.7	Independent t-test	t = 2.10	0.038*
Male n (%)	23 (51.1%)	26 (57.8%)	Chi-square test	χ² = 0.38	0.538
Comorbidities (%)	20 (44.4%)	18 (40.0%)	Chi-square test	χ² = 0.17	0.679
Mean Injury to Surgery Time (days)	4.2 ± 1.3	4.6 ± 1.5	Independent t-test	t = 1.33	0.188

Baseline data indicated that the average age was higher in the intertrochanteric group (66.1 vs. 61.5 years, p=0.038), whereas the proportions of genders and comorbidities were equal. The interval between time to surgery was also comparable among the groups, suggesting that both groups had similar preoperative conditions. Table 2 demonstrates the mobility outcomes after three and six months of surgery.

**Table 2:** Mobility Outcomes After Three and Six Months PROMIS-PF = Patient-Reported Outcomes Measurement Information System - Physical Function, TUG = Timed Up-and-Go, SD = Standard Deviation, CI = Confidence Interval, * = Significance at p-value <0.05

Outcome	Intertrochanteric Group Mean ± SD	Shaft Femur Group Mean ± SD	Test Value	Mean Difference	95% CI	p-value
PROMIS PF 10a (3 months)	58.4 ± 6.5	52.7 ± 7.2	t = 3.10	5.7	2.9 – 8.5	0.003*
PROMIS PF 10a (6 months)	66.3 ± 5.8	60.6 ± 6.7	t = 3.00	5.7	2.0 – 9.4	0.004*
TUG Test (3 months, sec)	18.2 ± 3.9	21.1 ± 4.5	t = -2.60	–2.9	–5.1 to –0.7	0.012*
TUG Test (6 months, sec)	14.7 ± 2.8	17.5 ± 3.6	t = -2.84	–2.8	–4.8 to –0.8	0.007*

In terms of mobility, the intertrochanteric fracture group had more favorable PROMIS-PF 10a scores after three- and six-month follow-ups (58.4 vs. 52.7, p=0.003, and 66.3 vs. 60.6, p=0.004). The TUG tests were lower in the intertrochanteric fracture group after both follow-ups (18.2 vs. 21.1 s, p=0.012; 14.7 vs. 17.5 s, p=0.007), indicating less disability in this group. Table 3 illustrates the complications of surgery and the length of hospital stay.

**Table 3:** Complications and Hospital Stay n = Number of participants, SD = Standard Deviation, % = Percentage, * = Significance at p-value <0.05

Parameter	Intertrochanteric Group	Shaft Femur Group	Test Used	Test Value	Significance (p-value)
Complications n (%)	5 (11.1%)	6 (13.3%)	Chi-square test	χ² = 0.11	0.743
Mean Hospital Stay (days) Mean ± SD	7.2 ± 2.1	9.0 ± 3.4	Independent t-test	t = -3.36	0.001*

The risk of complications was low and not significantly different across the groups (five (11.1%) vs. six (13.3%), p=0.743). The complications seen were mainly postoperative wound infections, transfusion requirements, and delayed union, with no major intraoperative events. The duration of hospital stay was shorter in the intertrochanteric fracture group (7.2 ± 2.1 vs. 9.0 ± 3.4 days, p=0.001), demonstrating shorter recovery time. Table 4 demonstrates the association of age with mobility outcomes.

**Table 4:** Correlation Between Age and Mobility Outcomes PROMIS-PF = Patient-Reported Outcomes Measurement Information System - Physical Function, TUG = Timed Up-and-Go, * = Significance at p-value <0.05

Variable Pair	Correlation Coefficient (r)	Statistical Test	Test Value (t)	p-value
Age vs. PROMIS PF 10a (3 months)	-0.18	Pearson's r	-1.71	0.091
Age vs. PROMIS PF 10a (6 months)	-0.15	Pearson's r	-1.42	0.159
Age vs. TUG Test (3 months)	0.21	Pearson's r	2.01	0.048*
Age vs. TUG Test (6 months)	0.19	Pearson's r	1.81	0.074

The correlation analysis found weak to moderate associations between age and mobility outcomes. Age demonstrated slightly positive relationship with TUG test time at three months (r=0.21, p=0.048), indicating that the older patients might be a little slower to finish the test. Nevertheless, age was not significantly associated with PROMIS-PF 10a scores at both time periods (p>0.05), which means that the better mobility results in the intertrochanteric group were not significantly affected by the age gap between groups. These results indicated improved early mobility and reduced hospital stay after intertrochanteric fracture fixation compared to shaft femur fractures.

## Discussion

The purpose of this study was to compare the early postoperative mobility patterns, complication rates, and length of hospital stay of patients admitted to a hospital where surgical fixation was used to treat intertrochanteric and shaft femur fractures. The findings indicated that the fracture location was a key determinant of recovery outcomes. The improved mobility of patients with intertrochanteric fractures can be attributed to the metaphyseal region's better vascular supply and biomechanical stability, which facilitates earlier weight-bearing [12]. Another study also corroborates this by showing that the stable fixation of intertrochanteric fractures can ensure a quicker transition to assisted ambulation [13].

In contrast, high-energy trauma causes shaft femur fractures, leading to increased damage to soft tissues and slowed recovery [14]. Correlation analysis between age and mobility outcomes confirmed that age had a slight influence on them (p>0.05 for PROMIS scores), indicating that the slower process of recovery was mainly due to the injury mechanism rather than age differences. Another study showed that in diaphyseal fractures, intramedullary nailing offers high stability, although early recovery can be limited by thigh pain, hip pain from the entry site, as well as by abductor weakness from gluteal disruption [15]. This study’s findings also align with the previous literature, which demonstrated that the early functional scores of intertrochanteric fracture patients were higher compared to shaft fractures treated with nailing [16]. In this study, the intertrochanteric group contained older people (62.3 ± 9.4 vs. 35.7 ± 11.2 years, p < 0.001) yet demonstrated better mobility, which suggested that fracture type influenced the recovery process more than age, though age could still be a confounder. The fact that the hospital stay was shorter in the intertrochanteric group coincides with the trends of recent enhanced recovery pathways, emphasizing early mobilization and discharge planning [17]. Similarly, another study observed that earlier mobilized patients incurred fewer inpatient days, indicating that functional gains equate to cost savings [18].

The non-significant complication rate across different groups was consistent with a study, which mentioned that the risk of infection and reoperation is low with both types of surgical fixations when performed by competent surgeons [19]. Another study also confirmed this, indicating that a surgeon’s expertise is more relevant than the type of implant [20]. The research advocates the importance of patient counseling for realistic expectations regarding mobility and discharging schedules based on the location of the fracture.

These findings had their own practical implications. Fracture-specific rehabilitation protocols should be applied along with earlier aggressive mobilization for intertrochanteric fractures, and graduated protocols should be applied for shaft fractures. Preoperative counseling should be set with realistic expectations based on the type of fracture. Future research should validate these protocols and examine long-term outcomes which are beyond six months.

The limitations of this study are its single-center design and a relatively small sample size, which can hinder generalizing the study results. The intertrochanteric group was older with a higher mean age which could be considered a confounding variable in the mobility results. In addition, the study did not measure functional recovery at six months, thus possibly not reflecting the effects of long-term recovery. Additional large multicenter trials should replicate these findings and compare the outcomes of intertrochanteric fractures managed with intramedullary nailing versus dynamic hip screw fixation in order to facilitate more individualized rehabilitation plans in the future.

## Conclusions

This study aimed to provide a comparison between postoperative mobility, complication rates, and length of stay in patients who were surgically treated having intertrochanteric and shaft of femur fractures. The results demonstrated that intertrochanteric patients reported a higher functional mobility - early walk speed and shorter hospital stays than shaft femur fracture patients.

These results support the fact that the type of fracture and treatment procedure affect the initial outcomes of rehabilitation. These dissimilarities in the two surgical procedures should be taken into consideration when a surgeon and the rehabilitation staff are advising patients and making an individual rehabilitative program.
